# Pre‐existing interstitial lung disease does not affect prognosis in non‐small cell lung cancer patients with PD‐L1 expression ≥50% on first‐line pembrolizumab

**DOI:** 10.1111/1759-7714.13725

**Published:** 2020-11-13

**Authors:** Ou Yamaguchi, Kyoichi Kaira, Shun Shinomiya, Atsuto Mouri, Kosuke Hashimoto, Ayako Shiono, Yu Miura, Tomoe Akagami, Hisao Imai, Kunihiko Kobayashi, Hiroshi Kagamu

**Affiliations:** ^1^ Department of Respiratory Medicine, Comprehensive Cancer Center Saitama Medical University International Medical Center Saitama Japan

**Keywords:** Immune checkpoint inhibitor, interstitial lung disease, non‐small cell lung cancer, pembrolizumab, pneumonitis

## Abstract

**Background:**

The safety of pembrolizumab monotherapy in treatment‐naïve non‐small cell lung cancer (NSCLC) patients with high programed death‐ligand 1 (PD‐L1) expression and pre‐existing interstitial lung disease (ILD) has not yet been determined. Here, we aimed to evaluate the prognosis, efficacy and safety associated with pembrolizumab in such settings.

**Methods:**

In this single‐institution retrospective study conducted from May 2017 to October 2019, pembrolizumab was administered to 72 Japanese patients with treatment‐naïve advanced NSCLC with PD‐L1 tumor proportion score (TPS) ≥50%. Patients with ILD were assigned to the ILD group, and those without to the non‐ILD group. Between‐group comparisons were then performed.

**Results:**

Of the 72 patients, 61 (84.7%) were male. The median age was 70 years. A total of 64 patients (88.9%) had a smoking history, median PD‐L1 TPS status was 77.5%, and 10 of the 72 patients (13.9%) had ILD on pretreatment computed tomography. The objective response rate (ORR) was 45.8% and disease control rate (DCR) was 75.0%. The ORR was 70.0% and DCR was 90.0% in the ILD group, while the ORR was 41.9% and DCR was 72.6% in the non‐ILD group. The median overall survival was 568 days; the value in the non‐ILD group was 521 days, while in the ILD group was not reached. There was no significant difference between the two groups (log‐lank, *P* = 0.73).

**Conclusions:**

Pembrolizumab was administered to patients with pre‐existing ILD with no difference in prognosis compared to patients without ILD. In patients with ILD, physicians should consider the expected long‐term prognosis and risk of adverse events.

## Graphical abstract

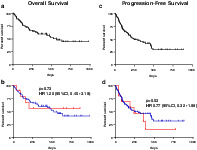

The safety and prognoses of pembrolizumab monotherapy in treatment‐naïve advanced NSCLC patients with high PD‐L1 expression and pre‐existing interstitial lung disease (ILD) has not yet been determined. In this retrospective study, the incidence of pneumonitis in patients with pre‐existing ILD was comparable to those without pre‐existing ILD. Moreover, this study showed that patients with pre‐existing ILD had comparable efficacy and prognoses compared to patients without pre‐existing ILD. When considering the use of pembrolizumab for NSCLC patients with pre‐existing ILD, physicians should consider the balance between safety and long‐term prognosis, which can be obtained.

## Introduction

Pembrolizumab is an antibody that exhibits significant antitumor effects by programed death‐1 (PD‐1) inhibition in advanced non‐small cell lung cancer (NSCLC).^1^ Pembrolizumab is associated with significant progression‐free survival (PFS) and overall survival (OS) improvements compared to conventional platinum‐based chemotherapy as a first‐line treatment in patients with a PD‐L1 tumor proportion score (TPS) ≥50%.^2^, ^3^ However, the use of PD‐1 inhibitors is known to cause immune‐related adverse events (irAEs) in multiple organs.^4^ Among them, pneumonitis is widely identified as an AE that can lead to death.^5^, ^6^

The occurrence frequency of drug‐induced pneumonitis in the treatment of advanced NSCLC differs according to the type of anticancer drugs. The incidence of pneumonitis caused by epidermal growth factor receptor‐tyrosine kinase inhibitor (EGFR‐TKI) in NSCLC patients harboring the *EGFR* mutation is 1% to 4%.^7^, ^8^, ^9^, ^10^, ^11^ Several clinical trials that compared PD‐1 inhibitors with other cytotoxic agents demonstrated that the incidence rates of pneumonitis were 2.1% to 6.8% in the case of PD‐1 inhibitor use and 0% to 2.0% in association with cytotoxic chemotherapy (Table S1).^2^, ^12^, ^13^, ^14^, ^15^, ^16^, ^17^, ^18^ Although the incidence of pneumonitis in cases with PD‐1 inhibitor use tends to be higher than that in cases receiving cytotoxic chemotherapy, the survival benefit associated with the administration of PD‐1 inhibitors has been elucidated by several clinical trials.

The incidence of pneumonitis in NSCLC patients previously treated with pembrolizumab monotherapy is 4% to 5%, and that in cases with grade 3 or higher disease is 2%.^12^ In patients initially treated with the agent, the value is 5.8% to 8%, and in grade 3 or higher cases it is 2.6% to 3%.^2^, ^18^ A Japanese subset analysis of the KEYNOTE‐024 trial reported that the frequency of pneumonitis onset was 9.5%, and that in grade 3 or higher cases it was 4.8%.^19^ The frequency of pembrolizumab‐induced pneumonitis tends to be higher in treatment‐naive patients than previously treated patients. In addition, the frequency may be higher in Japanese patients.

In a multicenter prospective cohort study of 138 NSCLC patients, Suzuki *et al*. reported that 20 patients (14.5%) developed pneumonitis after PD‐1 inhibitor treatment.^20^ Moreover, the percentage predicted forced vital capacity (FVC) reduction, percentage predicted forced expiratory volume in one second (FEV1) reduction, and modified Medical Research Council scale have been identified as risk factors of pneumonitis. In the real‐world setting, PD‐1 inhibitor‐induced pneumonitis may be more frequent than in clinical trials.

Nowadays, NSCLC patients with pre‐existing interstitial lung disease (ILD) are often excluded from prospective clinical trials; thus, data on the frequency of pneumonitis and its clinical course are often unknown in patients with pre‐existing ILD who received any anticancer drugs. Fujimoto *et al*. conducted a phase II study to evaluate the safety of nivolumab in previously treated NSCLC patients with mild idiopathic interstitial pneumonia (IIP).^21^ Pneumonitis was reported in two of 18 patients (11.1%), both of whom showed mild toxicity. The results of their study suggest that PD‐1 inhibitors may be safe for use in patients with mild ILD.

Although the incidence of pneumonitis related to pembrolizumab as a first‐, second‐ or further line treatment has been previously reported, it remains unclear whether pembrolizumab is tolerable in treatment‐naïve patients with a PD‐L1 TPS ≥50% who have pre‐existing ILD. Accordingly, in this study we sought to retrospectively investigate patients' medical records to elucidate the occurrence frequency of pembrolizumab‐induced pneumonitis and the associated clinical outcomes in such patients.

## Methods

### Patient information

We retrospectively examined data on patients with a histological confirmation of NSCLC with a PD‐L1 TPS ≥50%. From January 2017 to November 2019, 87 patients received pembrolizumab monotherapy as first‐line therapy, and a PD‐L1 TPS ≥50% was observed in 74. Of these 74 patients, two were excluded from the study due to their participation in another clinical trial. Finally, 72 patients were eligible for study participation. This single‐institution retrospective study was approved by the Institutional Review Board (approval number 19–157) of Saitama Medical University International Medical Center.

Data on the patients' characteristics, including those on age, sex, initial clinical stage, smoking history and index, Eastern Clinical Oncology Group (ECOG) performance status, histology, PD‐L1 TPS status (Dako, 22C3), history of radiation therapy before pembrolizumab, and pretreatment laboratory tests, namely those pertaining to lactate dehydrogenase, C‐reactive protein, Krebs von den Lungen‐6 (KL‐6), and antinuclear antibodies (ANAs) were obtained. Pulmonary function tests were conducted in accordance with American Thoracic Society/ European Respiratory Society standards,^22^ using FUDAC‐77 (Fukuda Denshi Co., Ltd., Tokyo Japan) package. The pulmonary function tests included FVC and diffusing lung capacity for carbon monoxide (DLco). The FVC and DLco values were calculated according to the proposal of the Japanese Respiratory Society.^23^ Pre‐existing ILD was diagnosed by three pulmonologists (A.M., S.S. and O.Y.) based on high‐resolution computed tomography (HRCT), using the criteria for fibrosing interstitial criteria according to the official American Thoracic Society (ATS)/ European Respiratory Society (ERS)/ Japanese Respiratory Society/ Latin American Thoracic Association statement: idiopathic pulmonary fibrosis (2011).^24^

### Treatment information

All patients received 200 mg pembrolizumab monotherapy every three weeks. Treatment continued until the progression of the disease or the development of unacceptable adverse events (AEs), or when either the patient or physician requested treatment discontinuation.

### Response and toxicity evaluation

Tumor response was evaluated according to the Response Evaluation Criteria in Solid Tumors (RECIST) version 1.1.^25^ Toxicity was graded according to the Common Terminology Criteria for Adverse Events version 4.0. The CT patterns noted in the cases with pneumonitis caused by pembrolizumab were classified according to the ATS*/*ERS international multidisciplinary classification used by Nishino *et al*. as: acute interstitial pneumonia (AIP)*/*DAD pattern, hypersensitivity pneumonia (HP) pattern, cryptogenic organizing pneumonia (COP) pattern, nonspecific interstitial pneumonia (NSIP) pattern and others.^26^

### Statistical analysis

Statistical analyses were performed using Prism 8 (GraphPad). Fisher's exact test and Student's *t*‐test were used to examine the association between two categorical variables. Chi‐square tests were used to examine the association between three categorial variables. Survival curves were estimated using the Kaplan‐Meier method. All *P*‐values were two‐sided, and *P* < 0.05 was considered statistically significant.

## Results

### Patient demographics

The patient characteristics are listed in Table 1. A total of 61 patients (84.7%) were male and 11 (15.3%) were female. The median age was 70 years (range, 47 to 86), and 64 patients (88.9%) had a smoking history. The mean PD‐L1 TPS was 77.5% (range, 50 to 100), and median observation period for all 72 patients was 312 days (range, 2 to 978).

**Table 1:** Demographics of pembrolizumab‐treated patients

Variable (*n* = 72)	All patients treated with pembrolizumab			
	(*n* = 72)	Pre‐existing ILD		*P*‐value
		ILD group	Non‐ILD group	
		(*n* = 10)	(*n* = 62)	
Sex Male/female	61/11	10/0	51/11	0.17
Age <75/ ≥ 75 years	51/21	6/4	45/17	0.32
Smoking history Yes/No	64/8	10/0	54/8	0.28
ECOG PS 0–1/2–3	49/23	7/3	42/20	0.60
Histology AD/ SQ/others	40/13/19	5/2/3	35/11/16	0.93
Stage IIIB/ IV/recurrence	6/55/11	1/9/0	5/46/11	0.35
PD‐L1 status, % 50 to 75/over 75	31/41	4/6	27/35	>0.99
Any radiation therapy before pembrolizumab^†^ Yes/No	24/48	2/8	22/40	0.28
Emphysema Yes/No	41/31	6/4	35/27	0.56
KL‐6 median (range), U/mL	424.5 (150–4464)	479 (246–4464)	378 (150–4142)	0.56
LDH median (range), U/L	209.5 (130–1192)	231 (170–786)	209 (130–1192)	0.84
CRP median (range), mg/dL	0.96 (0.02–23.38)	1.02 (0.07–12.52)	0.96 (0.02–23.38)	0.54

^†^ “Radiation therapy” includes both thoracic irradiation and extra‐thoracic irradiation.

AD, adenocarcinoma; CRP, C‐reactive protein; ECOG, Eastern Cooperative Oncology Group; ILD, interstitial lung disease; KL‐6, krebs von den lungen‐6; LDH, lactate dehydrogenase; PD‐L1, programed death‐ligand 1; PS, performance status; SQ, squamous cell carcinoma.

**Figure 1:**
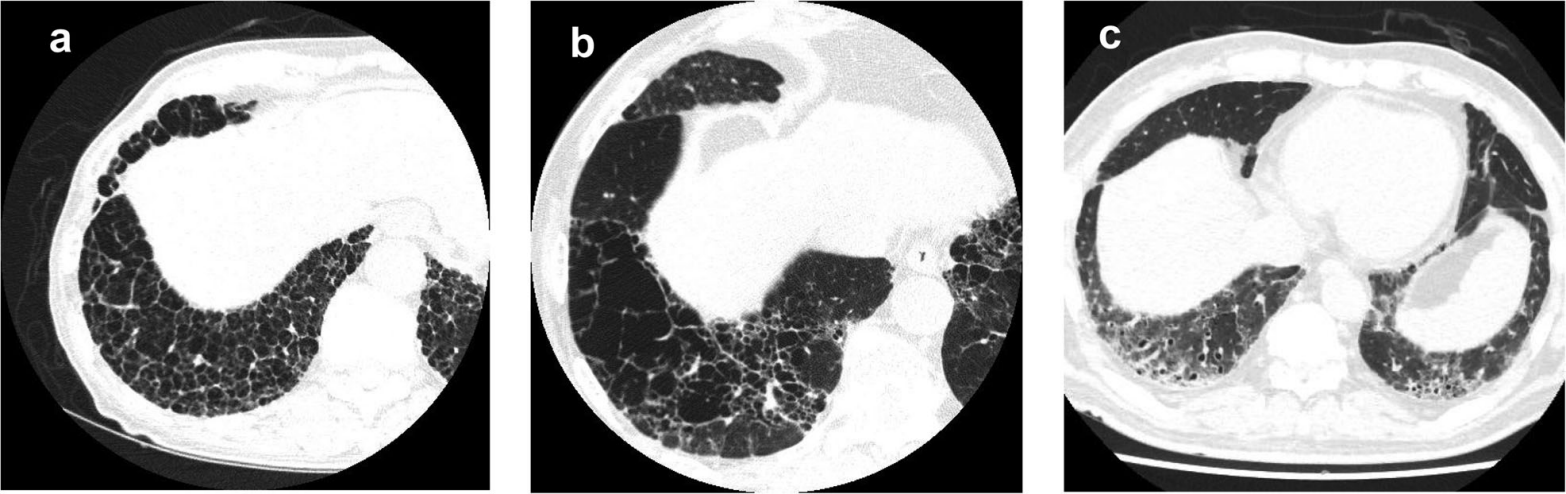
Background lung findings before treatment with pembrolizumab. (**a**) Computed tomography (CT) of the lung field in Case 1. Honeycomb changes were observed immediately below the pleura, and the patient was considered to have usual interstitial pneumonia (UIP). (**b**) CT image of Case 3. Reticular shadows and emphysema were observed mainly in the lower lobe. The honeycomb structure is not clear. This was determined to be possible UIP pattern. (**c**) CT image of Case 8. Reticulated shadow and extensive ground‐glass opacities (GGOs) were observed mainly in the dorsal lower lobe of both lungs. This was determined to be inconsistent with UIP pattern.

Pretreatment HRCT revealed ILD presence in 10 (13.9%) of 72 patients. Patients with pre‐existing ILD were assigned to the ILD group and those without it to the non‐ILD group. There were no significant differences in the patients' demographics between the groups. Fig 1 shows the representative CT images of the ILD group. Fig 1(a) shows the case of a patient with a honeycomb finding just below the pleura at the bottom of both lungs; we judged this ILD as having a UIP pattern. Fig 1(b) shows reticular shadows and emphysema were observed mainly in the lower lobe. On the other hand, since there was no honeycombing, we determined that it was a possible UIP pattern. Figure 1(c) shows the presence of extensive lung shadows at the bottoms of both lungs, which were superior to reticular shadows, and were judged as being inconsistent with UIP patterns.

The characteristics of the 10 patients with ILD are listed in Table 4. All patients in the ILD group were male and had a smoking history. As for the classification of ILD by HRCT, eight patients had possible UIP pattern, one patient had a UIP pattern and one patient had inconsistent with UIP pattern. The median %FVC was 103.5% and %DL_CO_ was 76.2%. Patients with inconsistent UIP patterns (Case 8) had not been diagnosed with collagen disease, but their ANA value was 320 times higher.

**Table 2:** Best response to pembrolizumab monotherapy in patients with PD‐L1 TPS ≥50%

Best response	All patients treated with pembrolizumab			*P‐*value
	% (*n* = 72)	Pre‐existing ILD		
		With ILD %	Without ILD %	
		(*n* = 10)	(*n* = 62)	
Partial response	45.8% (33)	70.0% (7)	41.9% (26)	0.17
Stable disease	29.2% (21)	20.0% (2)	30.6% (19)	0.71
Progressive disease	16.7% (12)	10.0% (1)	17.7% (11)	>0.99
Not evaluated	8.3% (6)	0.0% (0)	9.7% (6)	0.59
Disease control rate	75.0% (54)	90.0% (9)	72.6% (45)	0.43
Objective response rate	45.8% (33)	70.0% (7)	41.9% (26)	0.17

ILD, interstitial lung disease; PD‐1, programmed death‐1; TPS, tumor proportion score.

### Efficacy and survival analysis

**Figure 2:**
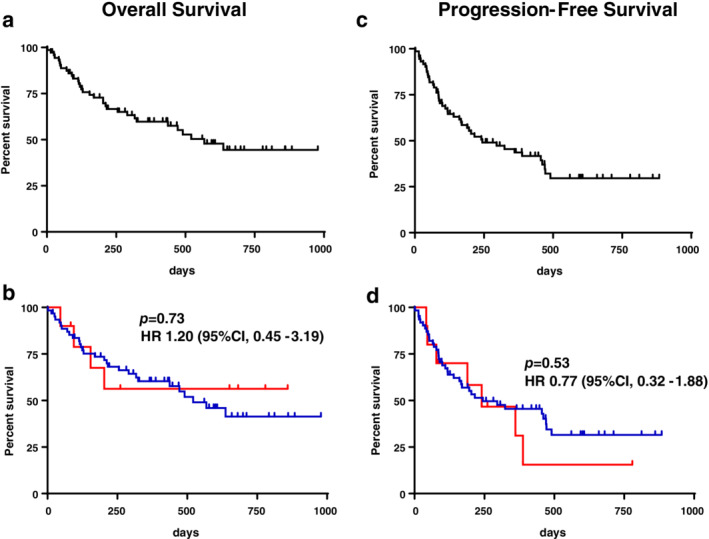
(**a**) Kaplan‐Meier curve of overall survival (OS) in 72 patients. (**b**) Kaplan‐Meier curve comparing the OS values between the interstitial lung disease (ILD) and non‐ILD groups. (**c**) Kaplan‐Meier curves of progression‐free survival (PFS) in 72 patients. (**d**) Kaplan‐Meier curve comparing the PFS values between the ILD and non‐ILD groups.
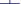
pre‐existing ILD−,
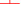
pre‐existing ILD+.

Table 2 shows the best response based on RECIST criteria. Of 72 patients, six (8.3%) were not evaluable, 33 (45.8%) had a partial response, 21 (29.2%) had stable disease, and 12 (16.7%) had progressive disease. In all 72 patients, the objective response rate (ORR) and disease control rate (DCR) were 45.8% (95% confidence interval [CI]: 34.3–57.3) and 75.0% (95% CI: 65.0–85.0), respectively. The ORR and DCR in the ILD group were 70.0% and 90.0%, respectively, whereas, those in the non‐ILD group were 41.9% and 72.6%, respectively, demonstrating no significant difference. Figure 2 shows the Kaplan‐Meier survival curves of the patients. The median overall survival (OS) and median progression‐free survival (PFS) duration for all patients were 568 days and 244 days, respectively. The OS in the non‐ILD group was 521 days, while survival was not achieved in the ILD group, demonstrating no significant difference (*P* = 0.73). The PFS in the non‐ILD group was 244 days, while that in the ILD group was 240 days, showing no significant difference (*P* = 0.53).

### Immune‐related adverse events

Table 3 shows the occurrence frequency of irAEs according to the presence or absence of ILD and non‐ILD. A total of 21 of the 72 patients (29.2%) had grade 3 or higher irAEs. Grade 3 or higher irAEs occurred in three patients in the ILD group (30.0%). A total of 16 of 72 patients (22.2%) developed pneumonitis due to pembrolizumab. Grade 3 or higher pneumonitis occurred in eight of these 72 patients (11.1%). Pneumonitis developed in two of 10 patients (20.0%) in the ILD group. Grade 3 or higher pneumonitis was observed in one patient (10.0%). Pneumonitis developed in 14 of 62 patients in the non‐ILD group (22.6%). Grade 3 or higher pneumonitis developed in seven patients (11.3%). There was no significant difference in the incidence of pneumonitis between the groups.

**Table 3:** Immune‐related adverse events associated with pembrolizumab monotherapy in patients with PD‐L1 TPS ≥50%

	All patients treated with pembrolizumab			*P*‐value
	(*n* = 72) (%)	Pre‐existing ILD		
		ILD group	Non‐ILD group	
		(*n* = 10) (%)	(*n* = 62) (%)	
Pneumonitis	16 (22.2%)	2 (20.0%)	14 (22.6%)	>0.99
Grade 1 or 2	8 (11.1%)	1 (10.0%)	7 (11.3%)	>0.99
Grade 3 or 4	8 (11.1%)	1 (10.0%)	7 (11.3%)	>0.99
Thyroid dysfunction^†^ (Grade 1 or 2)	19 (26.4%)	5 (50.0%)	14 (22.6%)	0.12
Liver dysfunction	11 (15.3%)	2 (20.0%)	9 (14.5%)	0.64
Grade 1 or 2	7 (9.7%)	1 (10.0%)	6 (9.7%)	>0.99
Grade 3 or 4	4 (5.6%)	1 (10.0%)	3 (4.8%)	0.46
Renal dysfunction^†^ (Grade 1 or 2)	6 (8.3%)	1 (10.0%)	5 (8.1%)	>0.99
Gastrointestinal toxicity (appetite loss, diarrhea, enteritis)	6 (8.3%)	1 (10.0%)	5 (8.1%)	>0.99
Grade 1 or 2	2 (2.8%)	1 (10.0%)	1 (1.6%)	0.26
Grade 3 or 4	4 (5.6%)	0 (0.0%)	4 (6.5%)	>0.99
Other grade 3 or 4 AEs	4 (5.6%)	1^‡^ (10.0%)	3^§^ (4.8%)	0.46
Any grade 3 or higher AEs	21 (29.2%)	3 (30.0%)	18 (29.0%)	>0.99

^†^ No grade 3 or higher AEs were observed.

^‡^ Encephalitis.

^§^ Fulminant type 1 diabetes, Amylase elevation, skin rash.

AE, adverse event; ILD, interstitial lung disease; PD‐1, programmed death‐1; TPS, tumor proportion score.

The CT pattern of pneumonitis due to pembrolizumab in the non‐ILD group yielded COP patterns in eight patients, HP patterns in two patients, AIP/DAD patterns in two patients, NSIP patterns in one patient, and other types in one patient. Nine of the 14 patients received systemic steroid therapy. The remaining five of 14 patients did not need steroid therapy and were only followed‐up. All 14 patients experienced improvements in the degree of pneumonitis.

All 16 patients who developed pneumonitis discontinued pembrolizumab once, but it was readministered in three patients after improvement of their pneumonitis. One of the three patients who received pembrolizumab again had a relapse of pneumonitis, but two patients had no relapse of pneumonitis. In addition, three of the 16 patients received chemotherapy as second‐line treatment after confirmation of exacerbation. Two patients received platinum combination chemotherapy and one patient received S1 monotherapy.

**Table 4:** Characteristics of patients with pre‐existing interstitial lung disease

Case	Age (years)	Sex	Histology	PD‐L1 status, Dako 22C3	Pattern of pre‐existing ILD on HRCT	Smoking status, pack‐years	Baseline KL‐6 value, U/mL	Baseline LDH level, U/L	Baseline ANA value, titer	%FVC, %	%DLco, %	Pneumonitis due to pemblolizumab, Yes/ No
1	67	Male	AD	70	UIP	60	675	393	40	73.4	45.5	No
2	79	Male	NOS	70	Possible UIP	108	465	274	40	119.4	55.3	No
3	75	Male	AD	95	Possible UIP	50	301	200	40	95.5	125.3	No
4	74	Male	NOS	90	Possible UIP	53	4464	198	<40	111.6	54.1	No
5	70	Male	NOS	90	Possible UIP	66	607	786	40	NA	NA	No
6	66	Male	SQ	90	Possible UIP	46	440	230	40	113.4	108.5	No
7	70	Male	AD	80	Possible UIP	50	340	377	40	106.0	77.4	Yes
8	64	Male	SQ	50	Inconsistent with UIP	64	635	195	320	88.8	NA	Yes
9	76	Male	AD	60	Possible UIP	42	246	170	<40	NA	NA	No
10	80	Male	AD	90	Possible UIP	27.5	493	232	40	88.7	76.2	No

AD, adenocarcinoma; ANA, antinuclear antibody; DLco, carbon monoxide diffusing capacity; FVC, forced vital capacity; HRCT, high resolution computed tomography; ILD, interstitial lung disease; KL‐6, Krebs von den Lungen‐6; LDH, lactate dehydrogenase; NA, not available; NOS, not otherwise specified; PD‐L1, programed death‐ligand 1; SQ, squamous cell carcinoma; UIP, usual interstitial pneumonia.

As an additional analysis, 16 patients with pneumonitis and 56 patients without pneumonitis were classified, and their efficacy and prognoses were compared. The ORR of the pneumonitis‐onset group was 43.8%. On the other hand, the ORR in the nononset group was 46.4%, and there was no significant difference between the two groups (*P* > 0.99). The DCR of the pneumonitis‐onset group was 87.5%. On the other hand, the ORR in the nononset group was 71.4%, and there was no significant difference between the two groups (*P* = 0.33). Figure S1 shows the Kaplan‐Meier curve comparing the pneumonitis‐onset group and the nononset group. The median PFS was 456 days (95% CI: 124–788) in the pneumonitis‐onset group and 203 days (95% CI: 63–343) in the nononset group, showing a good tendency in the pneumonitis‐onset group, although there was no significant difference (*P* = 0.37). The median OS was 637 days (95% CI: 306–968) in the pneumonitis‐onset group and 521 days (95% CI: 521–521) in the nononset group, showing a good tendency in the pneumonitis‐onset group (*P* = 0.65).

### Administration of pembrolizumab and subsequent therapies

Table 5 shows the administration status of pembrolizumab and the therapies used after pembrolizumab. At the time of the data cutoff, 15 of the 72 patients (20.8%) continued receiving pembrolizumab. In ILD group, two patients continued pembrolizumab treatment (20.0%), while in the non‐ILD group, 13 patients continued the treatment (21.0%). Of the 72 patients, 26 (29.2%) received any radiation therapy while receiving pembrolizumab or after the completion of pembrolizumab treatment. In the ILD group, six patients received any radiation therapy (60.0%), while in the non‐ILD group, the corresponding value was 20 patients (32.2%). Of the 72 patients, 15 (20.8%) received any subsequent treatment. In the ILD group, two patients received any subsequent therapy (20.0%); the corresponding value in the non‐ILD group was 13 patients (21.0%). Four patients in the non‐ILD group received atezolizumab as a second subsequent treatment.

**Table 5:** Administration status and subsequent therapies after use of pembrolizumab^†^

Number of patients (%)	All patients treated with pembrolizumab		
	(*n* = 72)	Pre‐existing ILD	
		ILD group (*n* = 10)	Non‐ILD group (*n* = 62)
Ongoing reception of pembrolizumab	15 (20.8%)	2 (20.0%)	13 (21.0%)
Number of doses of pembrolizumab, median (range)	4 (1–34)	4 (1–28)	4.5 (1–34)
Radiation therapy during or after pembrolizumab treatment	26 (36.1%)	6 (60.0%)	20 (32.2%)
Any subsequential therapies	15 (20.8%)	2 (20.0%)	13 (21.0%)
Number of regimens 1 2 3 4 ≧5	8 5 1 0 1	1 0 1 0 0	7 5 0 0 1
First subsequent therapies			
Received a first subsequent therapy Cytotoxic chemotherapy Platinum‐containing chemotherapy VEGF inhibitor	15 15 8 6	2 2 1 0	13 13 7 6
Second subsequent therapies			
Received second subsequent therapy Cytotoxic chemotherapy Platinum‐containing chemotherapy PD‐1 inhibitor (atezolizumab)	7 3 0 4	1 1 0 0	6 2 1 4

^†^ Data cutoff 20 November 2019.

PD‐1, programmed death‐1; ILD, interstitial lung disease; VEGF, vascular endothelial growth factor.

## Discussion

In this study, we found that the frequency of pneumonitis occurrence after pembrolizumab treatment was not significantly higher in patients with pre‐existing ILD than those without it, and the ORR tended to be higher in the ILD group than the non‐ILD group without statistical difference. To the best of our knowledge, this is a first retrospective study to identify the clinical aspects associated with the use of first‐line pembrolizumab monotherapy in NSCLC patients with pre‐existing ILD. However, further investigation is warranted to examine the efficacy and tolerability of first‐line pembrolizumab in NSCLC patients with a PD‐L1 TPS ≥50% according to the pre‐existing ILD level.

In general, it is known that any conventional cytotoxic chemotherapy regimen used for NSCLC patients with pre‐existing ILD is associated with a high incidence of drug‐induced pneumonitis or acute ILD exacerbation, with some prospective trials studies demonstrating the occurrence frequency to be 4.3% to 9.5%.^27^, ^28^, ^29^, ^30^ The presence of pre‐existing interstitial shadows on CT representing UIP patterns is a risk factor of acute exacerbation associated with cytotoxic chemotherapy.^31^ Moreover, it is well‐known that pre‐existing ILD increase the incidence of drug‐induced pneumonitis after the administration of EGFR‐TKIs, particularly gefitinib, for NSCLC patients harboring *EGFR* mutation.^32^

There is no consensus on the safety of PD‐1 inhibitors in NSCLC patients with pre‐existing ILD. In a large post marketing study of nivolumab, Kenmotsu *et al*. reported that the presence of an abnormal lung shadow, as judged by physicians on chest CT, was a risk factor for the development of pneumonitis due to nivolumab.^33^ On the other hand, Fujimoto *et al*. described that nivolumab treatment may be feasible in NSCLC patients with mild interstitial pneumonia in a prospective phase II study.^21^ In their study, mild interstitial pneumonia was defined by a predicted vital capacity (%VC) value ≥80% and an HRCT image showing a possible UIP pattern or that was inconsistent with UIP pattern. Thus, there is still no consensus on whether pre‐existing ILD could be a risk factor for pneumonitis. The mechanism of drug‐induced pneumonitis may differ between immune checkpoint inhibitors and conventional anticancer drugs. In our study, among 10 patients with ILD, one patient had a UIP pattern, but the remaining nine patients had a possible UIP pattern or inconsistent with UIP pattern. In addition, the median %FVC was 103.5%, which is close to the value observed in patients with mild IIP, as described by Fujimoto *et al*.^21^ Our results suggest that pre‐existing ILD does not significantly increase the occurrence of drug‐induced pneumonitis caused by PD‐1 inhibitor.

In our study, the efficacy of pembrolizumab in patients with ILD tended to be higher than that in those without it. Additionally, as all the patients with ILD had a smoking history, the etiology of ILD may be closely related to smoking history. Tumor mutation burden (TMB) is a promising biomarker in the prediction of immune checkpoint inhibitor (ICI) efficacy. A recent phase III subset analysis reported favorable PFS values after nivolumab treatment in patients with a high TMB based on missense mutations, as obtained by whole exome analysis.^15^ TMB plays a crucial role in the determination of the cancer antigen source, and NSCLC is associated with strong immunogenicity.^34^ Smoking is the leading cause of TMB development in lung cancer.^35^ Moreover, smoking history was identified as a good prognostic factor in a subset analysis of CheckMate 057.^14^ Considering this evidence, the presence of ILD related to smoking history may be clinically identified as a favorable prognostic factor in the prediction of the therapeutic efficacy of PD‐1 inhibitors.

In our study, it is noteworthy that the incidence of pneumonitis was 20.0% in the ILD group and 22.6% in the non‐ILD group, and the incidence of grade 3 or higher pneumonitis was almost similar in the ILD group (10.0%) and non‐ILD group (11.3%). The incidence of other irAEs did not tend to be predominantly higher in the ILD group.

In terms of prognoses, the PFS tended to be slightly worse in the ILD group than the non‐ILD group, but there was no difference in the OS. There was also no significant difference between the groups in the number of pembrolizumab cycles and administration status of the subsequent treatment. In the ILD group, 60.0% of the patients received radiation therapy during or after pembrolizumab treatment, which may have contributed to the long‐term prognosis.^36^, ^37^

Our study has several limitations. First, our sample size was small, biasing the results of our evaluation. Second, the number of patients in the ILD and non‐ILD groups was not well‐balanced. Therefore, it may be difficult to compare the prognosis associated with pembrolizumab between the groups. In our study, three qualified pulmonologists concisely judged the presence of pre‐existing ILD, and the radiologic diagnosis of pembrolizumab‐induced pneumonitis was also strictly performed.

In conclusion, the use of pembrolizumab in first‐line treatment was useful and tolerable in advanced NSCLC patients with a PD‐L1 TPS ≥50%, regardless of the presence of pre‐existing ILD. According to the results of our study, the therapeutic benefit in patients with ILD seemed to be similar to that without ILD. We determined that if the patients with ILD were excluded from receiving ICIs, their survival may be shorter. The merit of our investigation encourages physicians considering the usefulness of ICI in patients with ILD.

## Disclosure

O. Yamaguchi received speaker honoraria from Ono Pharmaceutical Co., Ltd., Bristol‐Myers Squibb, Taiho Pharmaceutical, MSD, Chugai Pharmaceutical Co., Ltd., and AstraZeneca. K. Kaira received speaker honoraria from Ono Pharmaceutical Co., Ltd., Bristol‐Myers Squibb, Taiho Pharmaceutical, Chugai Pharmaceutical Co., Ltd., Eli Lilly Japan, and AstraZeneca. A. Mouri received speaker honoraria from Ono Pharmaceutical Co., Ltd., Bristol‐Myers Squibb, Taiho Pharmaceutical, MSD, Chugai Pharmaceutical Co., Ltd., and AstraZeneca. K. Kobayashi received research grants from AstraZeneca and Bristol‐Myers Squibb. K. Kobayashi received speaker honoraria from AstraZeneca, Pfizer Japan Inc., Ono Pharmaceutical Co., Ltd., Chugai Pharmaceutical Co., Ltd., and Bristol‐Myers Squibb. H. Kagamu received research grants from Novartis Pharma K.K., Nippon Boehringer Ingelheim Co., Ltd., Chugai Pharmaceutical Co., Ltd., Taiho Pharmaceutical, and Ono Pharmaceutical Co., Ltd. H. Kagamu received speaker honoraria from Chugai Pharmaceutical Co., Ltd., AstraZeneca, Pfizer Japan Inc., Eli Lilly Japan, Novartis Pharma K.K., Nippon Boehringer Ingelheim Co., Ltd., Ono Pharmaceutical Co., Ltd., Bristol‐Myers Squibb Co., Taiho Pharmaceutical, and MSD. The rest of the authors declare that there are no conflicts of interest.

## Supporting information


**Figure S1** Kaplan‐Meier curve analysis of prognoses in the pneumonitis onset group (*n* = 16) and nononset group (*n* = 56). (a) overall survival; (b) progression‐free survival.

**Table S1** Incidence rate of pneumonitis due to PD‐1 inhibitor use in clinical trials of advanced non‐small cell lung cancer.
