# Supplementary figures and images for: Pre‐existing interstitial lung disease does not affect prognosis in non‐small cell lung cancer patients with PD‐L1 expression ≥50% on first‐line pembrolizumab
Source: Thorac Cancer. 2020 Nov 13;12(3):304–13. doi: 10.1111/1759-7714.13725 (PMC7862785; doi:10.1111/1759-7714.13725)

## Slide 1
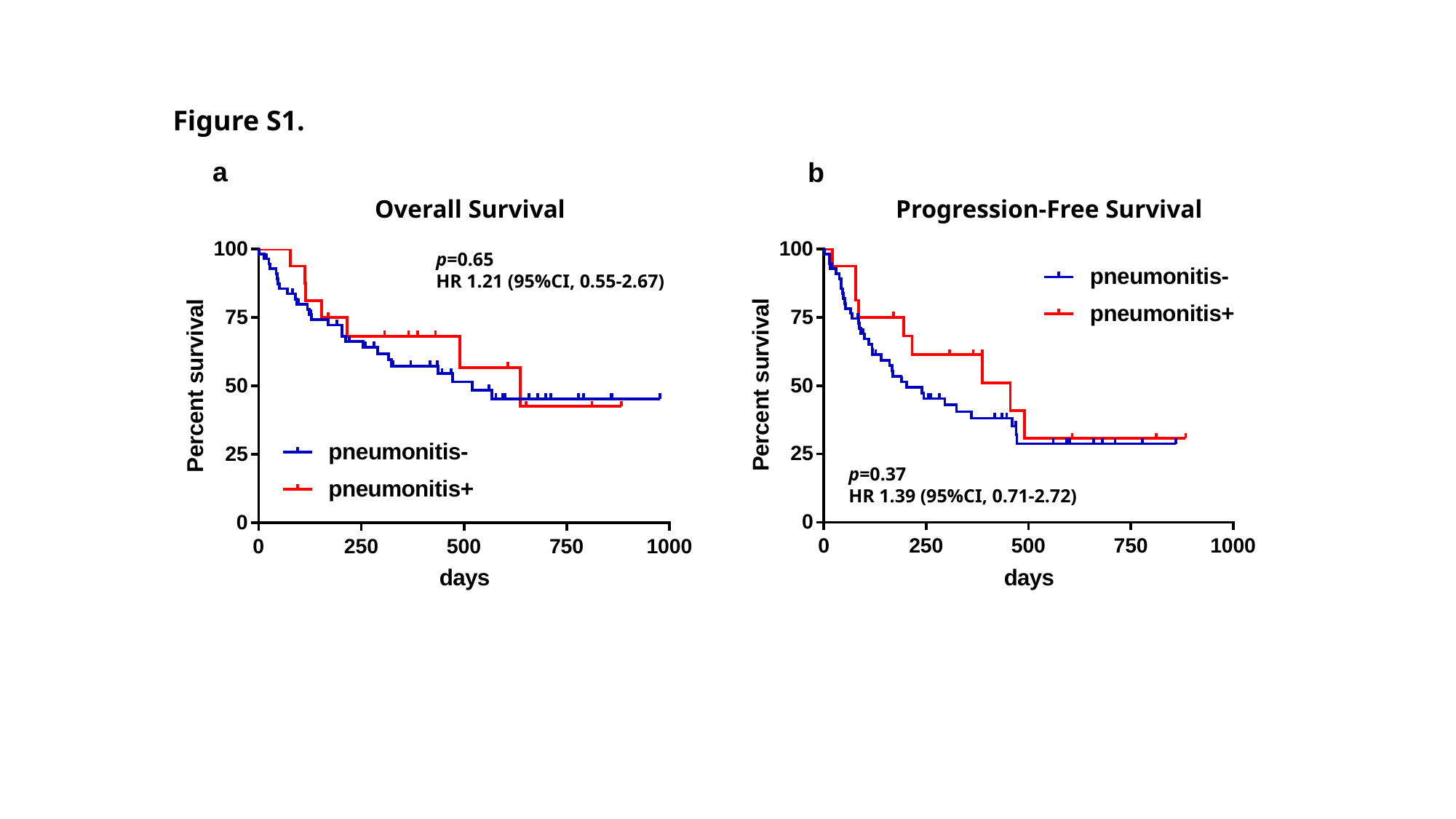

Figure S1.
a
b
Overall Survival
Progression-Free Survival
p=0.65
HR 1.21 (95%CI, 0.55-2.67)
p=0.37
HR 1.39 (95%CI, 0.71-2.72)

Supplement: Supplementary file 1 — Figure S1 Kaplan‐Meier curve analysis of prognoses in the pneumonitis onset group (n = 16) and nononset group (n = 56). (a) overall survival; (b) progression‐free survival. [file TCA-12-304-s001.pptx]
