# Supplementary material for: Pre‐existing interstitial lung disease does not affect prognosis in non‐small cell lung cancer patients with PD‐L1 expression ≥50% on first‐line pembrolizumab
Source: Thorac Cancer. 2020 Nov 13;12(3):304–13. doi: 10.1111/1759-7714.13725 (PMC7862785; doi:10.1111/1759-7714.13725)
Supplement: Supplementary file 2 — Table S1 Incidence rate of pneumonitis due to PD‐1 inhibitor use in clinical trials of advanced non‐small cell lung cancer. [file TCA-12-304-s002.docx]

**Table S1. Incidence rate of pneumonitis due to PD-1 inhibitor use in clinical trials of advanced non-small cell lung cancer**

| Clinical trial | PD-1 inhibitor | Status of treatment | N | Any grade, % | ≥Grade 3, % | Number of references |
| --- | --- | --- | --- | --- | --- | --- |
| KEYNOTE-024 | Pembrolizumab | Treatment-naïve | 154 | 5.8 | 2.6 | 2 |
| KEYNOTE-042 | Pembrolizumab | Treatment-naïve | 636 | 6.8 | 3.1 | 18 |
| KEYNOTE-010 | Pembrolizumab (2 mg/kg) | Previously treated | 339 | 4.7 | 2.1 | 12 |
|  | Pembrolizumab (10 mg/kg) |  | 343 | 4.4 | 2.0 | 12 |
| CheckMate 017 | Nivolumab | Previously treated | 131 | 4.6 | 0.0 | 13 |
| CheckMate 057* | Nivolumab | Previously treated | 287 | 2.1 | 1.4 | 14 |
| CheckMate 026 | Nivolumab | Treatment-naïve | 267 | 2.6 | 1.5 | 15 |
| CheckMate 078* | Nivolumab | Previously treated | 337 | 5.0 | 2.1 | 17 |
| OAK | Atezolizumab | Previously treated | 609 | 2.3 | 0.8 | 16 |

PD-1, programed death-1

*The incidence frequency was calculated by summing “pneumonitis” and “interstitial lung disease”.
